# Fine Particulate Air Pollution, Public Service, and Under-Five Mortality: A Cross-Country Empirical Study

**DOI:** 10.3390/healthcare8030271

**Published:** 2020-08-14

**Authors:** Siming Liu, Qing Wei, Pierre Failler, Hong Lan

**Affiliations:** 1 School of Statistics, University of International Business and Economics, Beijing 100029, China; liusiming@uibe.edu.cn (S.L.); wxrhqhd@163.com (Q.W.); 2 Economics and Finance Subject Group, Portsmouth Business School, University of Portsmouth, Portsmouth PO1 3DE, UK; 3 School of Economics and Management, Beijing Forestry University, Beijing 100083, China; honglan510@bjfu.edu.cn

**Keywords:** PM_2.5_, under-five mortality, public service, cross-country, heterogeneous effects, panel threshold model

## Abstract

The impacts of fine particulate matter (PM_2.5_) air pollution on health outcomes, especially those of children, have attracted worldwide attention. Based on the PM_2.5_ concentration data of 94 countries, including the least developed countries estimated by satellite observations in nearly 20 years, this paper investigated the impacts of PM_2.5_ pollution on under-five mortality rate (U5MR) and analyzed the role of public service in moderating the PM_2.5_-mortality relationship. Results indicated that PM_2.5_ pollution had significantly positive influence on U5MR globally. However, the effects of fine particulate pollution on child mortality were heterogeneous in terms of their significance and degrees in countries with different levels of development. A further test based on panel threshold model revealed that public service, measured by public education spending and sanitation service, played a positive moderating role in the PM_2.5_-mortality relationship. Specifically, when the ratio of public education expenditure in GDP of a country exceeded the first threshold value 3.39% and the second threshold value 5.47%, the magnitude of the impacts of PM_2.5_ pollution on U5MR significantly decreased accordingly. When the percentage of population with access to improved sanitation facilities in a country was over 41.3%, the health damaging effects were reduced by more than half. This paper fills the current gap of PM_2.5_ research in least developed countries and provides key policy recommendations.

## 1. Introduction

Air pollution, especially fine particulate, has been a significant global public health issue, attracting worldwide attention [1,2,3]. In recent years, many countries in the world (especially large developing ones such as China and India) have frequently suffered from haze or smog episodes characterized by high fine particulate matter (less than and equal to 2.5 μm in aerodynamic diameter, i.e., PM_2.5_). PM_2.5_, as one of the world’s primary air pollutants, is regarded as a pollutant that poses more danger to human health than ground-level ozone and other common air pollutants [4]. Simultaneously, an estimation released by the World Health Organization (WHO) indicates that more than 90% of the world’s population lived in places where the levels of PM_2.5_ concentrations exceed WHO limits (i.e., 10 μg/m^3^ in annual mean) in the year 2012 [5]. In this context, the health impacts of PM_2.5_ pollution have gained extensive academic attention from different fields such as medical science, public health, and economics [6,7,8,9,10,11,12,13].

A considerable volume of studies, especially those from the medical and public health fields, have empirically analyzed the influence of fine particulate air pollution on health outcomes, mainly in developed countries. The existing literature generally investigates the influence of PM_2.5_ pollution on hospital admission due to respiratory and various types of mortality (e.g., all-cause mortality, cardiopulmonary, lung cancer mortality) of residents in a particular country or specific cities in a country. Most of the studies have confirmed the adverse health effects of PM_2.5_ pollution [6,7,8,9,10,11]. Using the data of 500,000 adults in 51 cities of the United States, Pope III et al. [6] found that long-term exposure to PM_2.5_ pollution is a primary risk factor for cardiopulmonary and lung cancer mortality. Yorifuji et al. [7] discovered that acute exposure to PM_2.5_ is associated with increased risk of infant mortality in Tokyo, Japan. Additionally, Boldo et al. [8] and Pascal et al. [9] demonstrated the health-damaging effects of short- and long-term exposure to particulate matter air pollution in Spain and France, respectively. A fraction of studies showed that there are little or no statistically significant associations between fine particulate air pollution and health status [14,15].

Compared to the large volume of studies in developed countries, research that focuses on the PM_2.5_ pollution and health nexus of non-developed countries, especially of the least developed countries, is relatively scarce. Such studies in the literature could mostly be identified for a small handful of economies in transition and developing economies such as China, Brazil, etc. (According to the United Nations [16], all countries in the world can be classified as developed economies, economies in transition, developing economies, and least developed countries). Based on the data of 160 communities of 27 provinces in China, Li et al. [17] verified the existence of a positive correlation between PM_2.5_ and mortality. Utilizing the data of five urban city districts and two rural counties in the Beijing, Tianjin, and Hebei provinces of China in 2013, Zhou et al. [18] found that significant and positive relationships exist between PM_2.5_ concentration and mortality in rural areas, while insignificant association were observed in urban areas. Mantovani et al. [19] demonstrated a significantly positive relationship between exposure to PM_2.5_ and hospital admissions due to cardiovascular diseases in São Jose do Rio Preto, Brazil. Due to the data availability of PM_2.5_ pollution, there are very few studies documenting the relationship between PM_2.5_ pollution and health outcomes in low- and middle-income countries especially in the least developed countries. PM_2.5_ pollution remains an under-recognized environmental health risk in such countries [18]. Applying logistic and Poisson regression models, Egondi et al. [20] found that exposure to high levels of PM_2.5_ pollution is associated with a high risk for childhood morbidity and a high children mortality rate in Nairobi, Kenya. Using cross-country level data of Africa from 2000 to 2015, Owili et al. [21] provided the evidence that there exists a significant relationship between PM_2.5_ and under-five and maternal mortality in Africa.

In addition to focusing on the health effects of air pollution, there are a growing number of empirical studies especially those from economic fields paying attention to the issue of how to reduce the air pollution health risks by policy interventions. However, most of the work focuses on policy design, targeting declining air pollution from the source, by investigating the socioeconomic determinants of air pollution or addressing the health benefits of air pollution reduction by introducing air quality policies [22,23,24]. Few studies have empirically explored the factors influencing the pollution-health relationship. Generally speaking, the policies targeting reducing air pollution are supposed to play a dominant role in reducing the adverse effects of pollution and the results of many research articles indicate that imposing stringent environmental protection policies is beneficial in reducing air pollution and mortality [22,23]. Nevertheless, by quantifying the policy effects of the adoption of low emission zones (LEZs) on pollution reduction and health improvement in Germany, Gehrsitz [25] pointed out that the introduction of a LEZ can help to moderately reduce PM_10_ levels. However, such reduction does not significantly translate into improvement in health outcomes. In this sense, besides policies targeting declining PM_2.5_ pollution from the source, it is necessary to take account multiple policy instruments by probing the key factors (e.g., public service) conducive to reducing the health-damaging effects of PM_2.5_ pollution, especially for non-developed countries (Many non-developed countries are in the phase of rapid industrialization and urbanization, which is inevitably characterized by high energy consumptions and emissions to a large extent [26]).

Since the epithelial linings of children’s lungs and immune systems are not fully developed, children are more vulnerable to ambient air pollution compared to adults [20,27,28]. This paper focuses on evaluating the impacts of fine particulate air pollution on U5MR, as well as explores the role of public service in moderating the PM_2.5_-mortality relationship with panel data of 94 countries over the period 1998–2014. The contributions of this study to the empirical literature are as follows.

First, this paper examines the heterogeneous effects of fine particulate air pollution on U5MR in various types of countries under different development levels. Different from existing studies mostly investigating the health effects of PM_2.5_ pollution at the individual level in specific developed countries or in few non-developed large countries, besides the full sample analysis of 94 countries, this paper further compares the heterogeneous health effects of PM_2.5_ pollution among developed countries, economies in transition and developing economies, and least developed countries. This provides extensive evidence of PM_2.5_ pollution effects on children’s health from a more general perspective.

Secondly, this study explores the factors moderating the PM_2.5_-mortality link. Numerous studies have investigated the linear relationship between PM_2.5_ pollution and health outcomes, but very few empirical studies have paid attention to how to reduce the health-damaging effects of air pollution based on nonlinear analysis between the two. This paper expands this field by testing whether the impacts of PM_2.5_ pollution on children’s health are related to the performance of a country’s pubic service measured by public education spending and the condition of sanitation service.

Finally, this paper estimates the threshold values and threshold effects of PM_2.5_ pollution on U5MR. Utilizing panel threshold model, this paper verifies a single threshold effect and a double threshold effect between PM_2.5_ pollution and U5MR by selecting sanitation service and public education spending, respectively. When the values of a country’s public education spending and sanitation service lie in various regimes, the impacts are found to differ significantly.

**Figure 1:**
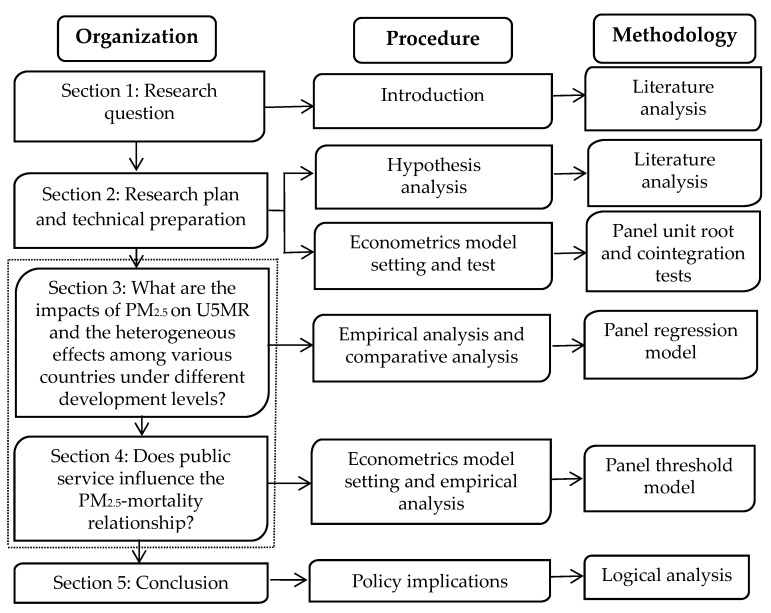
The logical framework of the study.

The paper is organized as follows: the next section presents the research plan and technical preparation. Empirical results regarding the influence of fine particulate air pollution on U5MR are reported in Section 3. The results of public service affecting PM_2.5_-mortality link are provided in Section 4. Section 5 provides the concluding remarks. The logical framework of the current study is shown in Figure 1.

## 2. Research Plan and Technical Preparation

### 2.1. Research Hypothesis

According to the core research questions in this paper, three hypotheses are introduced accordingly.

As discussed above, although the health effects of fine particulate air pollution in non-developed countries, especially in least developed countries, are rarely addressed, most of the studies have verified the significant positive relationship between PM_2.5_ pollution and related mortality and morbidity in the context of different countries or regions. Meanwhile, PM_2.5_ is one of the primary air pollutants in the world and poses severe danger to human health even in low concentrations. What is more, children compared to adults are more susceptible to the poor air quality [29]. Therefore, we formulated the following hypothesis:

**Hypothesis** **1** **(H1).** *A positive relationship exists between fine particulate air pollution and U5MR globally.*

Although fine particulate air pollution is supposed to positively affect U5MR globally, the health-damaging effects may differ among various countries under different socioeconomic development levels. On the one hand, the extent to which an individual is affected by air pollution principally depends on the duration of exposure and the concentration of the chemicals [30,31,32]. People who live in regions with severe air pollution will face the influence of accelerated depreciation of health capital stocks [12,33]. Overall, as PM_2.5_ pollution in countries with low socioeconomic development levels is more severe than those with high socioeconomic development levels, it is expected that the adverse health effects of fine particulate air pollution will be larger in the former countries. On the other hand, empirical studies, especially those at micro dimension, revealed that populations with lower socioeconomic status (e.g., low income, poor education) tend to have higher exposure to air pollution as well as less access to health services and thus suffer greater adverse health effects accordingly [34,35,36,37]. In this sense, this paper believes that the negative impacts of fine particulate air pollution on children’s health outcomes should be larger in the countries which are of lower socioeconomic development levels. Thus, it was reasonable to hypothesize that:

**Hypothesis** **2** **(H2).** *The effects of fine particulate air pollution on U5MR in the countries with low socioeconomic development levels are larger than those with high socioeconomic development levels.*

Theoretically, the effects of fine particulate air pollution on children’s health are related to the condition of a country’s public service (Due to data availability, this paper focuses on evaluating the role of public education and sanitation services in moderating the PM_2.5_-mortality link). This is mainly because improving the performance of public service is an effective way to enhance health capital stocks [38], which is conducive to alleviating the hazardous health effects of PM_2.5_ pollution. Specifically, increasing public education spending will generally improve people’s educational attainment and thus help the public (e.g., children’s parents) acquire more environmental and health knowledge, gain a clear understanding of the health-damaging effects of PM_2.5_ pollution, reduce the exposure to PM_2.5_ pollution, improve ability to manage children’s health, and gain more access to health care [39]. Therefore, improving public education spending is expected to reduce the adverse influence of PM_2.5_ pollution on children’s health. In addition, as pointed out by the WHO [40], the areas with weak health infrastructure (mostly in developing countries) will be the least able to cope without assistance to prepare and respond to the health risks due to climate change. This study believes sanitation services should also be an important factor influencing PM_2.5_-mortality relationship. As a sound sanitation service system will be beneficial to reduce the hazardous health effects by effective public health prevention and treatment of diseases associated with PM_2.5_ pollution. Finally, though no known studies have examined the role of public service in moderating the PM_2.5_-mortality link, the empirical results of Lu and Qi [38] indicate that the improvements in public service can reduce the adverse influence of PM_10_ on public health. Accordingly, we proposed the following hypothesis:

**Hypothesis** **3** **(H3).** *The effects of fine particulate air pollution on U5MR will decrease with the rise of a country’s public education spending and sanitation service improvement.*

### 2.2. Baseline Model

In the seminal work on the economics of health status, Grossman [41] proposed a theoretical production framework to model the demand for health. The Grossman health production function, which describes the relationship between individual health input and output, is initially designed for analyzing health production at the micro level [12,42]. At present, the health production framework has also been widely applied in numerous empirical studies at the macro level without losing theoretical ground [12,42,43,44,45,46]. Following the studies of Chen et al. [44], Feng et al. [12], Fotourehchi [43], and Lu and Qi [38], this paper believes that children’s health outcomes of a country are mainly affected by environmental, economic, social, educational and health care factors. Correspondingly, the health production function at the macro level in this study can be formulated as follows:

(1) H=F(Env,Eco,Soc,Edu,HC)

where *H* donates children’s health status, measured by U5MR. *Env*, *Eco*, *Soc*, *Edu,* and *HC* refer to environmental, economic, social, educational, and health care factors, respectively.

On the basis of Equation (1), the econometrics models described in Equation (2) were employed to detect the association between fine particulate air pollution and U5MR (As U5MR, urbanization level and technological innovation capacity are in the form of proportion, the current study did not deal with these variables logarithmically [46,47]).

(2) $$\begin{array}{c} U5{MR}_{it}=c+\delta \ln{PM}_{it}+\beta_{1}\ln E{co}_{it}+\beta_{2}\ln{PD}_{it}+\beta_{3}{Urb}_{it}+\beta_{4}In{no}_{it}+\\ \beta_{5}\ln Edu_{it}+\beta_{6}\ln HE_{it}+\alpha_{i}+\lambda_{t}+\varepsilon_{it} \end{array}$$

where *U5MR_it_* represents under-five mortality rate for country *i* and year *t*. *PM* refers to fine particulate air pollution level. *Eco*, *PD, Urb*, *Inno, Edu*, and *HE* are a set of control variables, denoting economic development level, population density, urbanization level, technological innovation capacity, education level, and health care expenditure, respectively. $\alpha_{i}$ and $\lambda_{t}$ are included in the model specification to control the country- and time-specific effects. *ε* is a disturbance term.

### 2.3. Variable and Data

#### 2.3.1. Variable Measurement

In this subsection, the measurement of the dependent variable and the core independent variable are initially introduced. Then, the selection and measurement of control variables in this paper are described.

**Under-five mortality rate.** As one of the UN Millennium Development Goals (MDGs), U5MR is widely used to measure children’s health outcomes and national health status for studies at the macro level especially at the country level. U5MR is calculated by the probability of dying by age 5 per 1000 live births in a country.

**Fine particulate air pollution.** Consistent with most studies in the literature [48,49,50], in this study, PM_2.5_ concentrations were selected as the index of fine particulate air pollution. Overall, there are two types of methods, i.e., population-weighted and geographic-mean, to estimate the PM_2.5_ concentrations on the regional or national scale. The population-weighted PM_2.5_ concentrations are calculated based on the spatial distribution of total population exposure, which should be superior to assess the influence of fine particulate air pollution on children’s health [51,52]. Therefore, we mainly employed population-weighted annual mean PM_2.5_ concentrations of each country to represent the level of fine particulate air pollution. Meanwhile, the geographic-mean PM_2.5_ concentration was given for robustness tests.

**Control variables.** The control variables in this paper were primarily selected from economic, social, education, and health care dimensions. Economic development level, measured by GDP per capita, is widely regarded as an unneglectable factor which affects the health outcomes at the macro level [12,42,43,44]. Following the work of Fotourehchi [43] and Lu and Qi [38], variables representing social factors include population density, urbanization level, and technological innovation capacity, which are measured by the number of people living in each unit of area, the share of urban population in total and the ratio of research and development (RD) expenditure to GDP, respectively. The education factor is identified as an important determinant of health status [12,41,45]. In this paper, mean years of schooling was employed as a proxy for educational levels. According to previous studies [43,44,46], the health expenditure is crucial to explaining the health outcomes in a certain region. In this paper, health expenditure per capita was employed to represent the input of health care in a country.

#### 2.3.2. Data and Descriptive Statistics

Due to data availability, the data of 94 countries from 1998 to 2014 were studied in this paper. According to United Nations [16], the sample countries selected in this paper are classified as 36 developed economies (i.e., Australia, Austria, Belgium, Bulgaria, Canada, Croatia, Cyprus, Czech, Denmark, Estonia, Finland, France, Germany, Greece, Hungary, Iceland, Ireland, Italy, Japan, Latvia, Lithuania, Luxembourg, Malta, Netherlands, New Zealand, Norway, Poland, Portugal, Romania, Slovakia, Slovenia, Spain, Sweden, Switzerland, United Kingdom and United States), 9 economies in transition (i.e., Armenia, Azerbaijan, Belarus, Kazakhstan, Kyrgyzstan, Moldova, Serbia, Tajikistan and Ukraine), 39 developing economies (i.e., Algeria, Argentina, Bolivia, Brazil, Chile, China, Colombia, Costa Rica, Ecuador, Egypt, Guatemala, Honduras, India, Indonesia, Iran, Jamaica, Kenya, Korea, Macedonia, Malaysia, Mauritius, Mexico, Morocco, Nicaragua, Pakistan, Panama, Paraguay, Peru, Philippines, Saudi Arabia, Salvador, Singapore, South Africa, Thailand, Trinidad and Tobago, Tunisia, Turkey, Uruguay and Vietnam) and 10 least developed countries (i.e., Cambodia, Congo, Ethiopia, Mozambique, Myanmar, Nepal, Senegal, Sudan, Tanzania, Zambia).

In terms of data source, except for average educational attainment and PM_2.5_ concentrations, the raw data on all other variables were obtained from World Development Indicators (WDI) database compiled by the World Bank [53]. The data of mean years of schooling in each country were collected from the United Nations Educational, Scientific, and Cultural Organization (UNESCO) [54]. 

Since the PM_2.5_ concentrations have not been monitored in many countries, especially in the least developed countries with low socioeconomic development levels, the data of PM_2.5_ concentrations could not be obtained through official channels. In this context, the Atmospheric Composition Analysis Group at Washington University estimated the data of PM_2.5_ concentrations at the country level since 1998 based on satellite observations. In this paper, data on both population-weighted and geographic-mean annual mean PM_2.5_ concentrations were taken from the Atmospheric Composition Analysis Group at Washington University [55], which was estimated by combining aerosol optical depth (AOD) with the GEOS-Chem chemical transport model and subsequently calibrated to global ground-based observations of PM_2.5_ using geographically weighted regression [3,51,56,57]. A subset of the global PM_2.5_ concentrations data at a 0.1 × 0.1 resolution and geographically weighted regression adjustment, which has been widely employed in numerous previous studies at the regional or national level [57,58,59], was utilized in this paper.

In addition, to ensure the comparability of the data, GDP per capita and health expenditure per capita of each country were measured at the 2010 constant dollar. The linear interpolation imputation method was mainly adopted to replace the missing values for a small number of sample countries. The statistical information of dependent and independent variables is given in Table 1.

**Figure 2:**
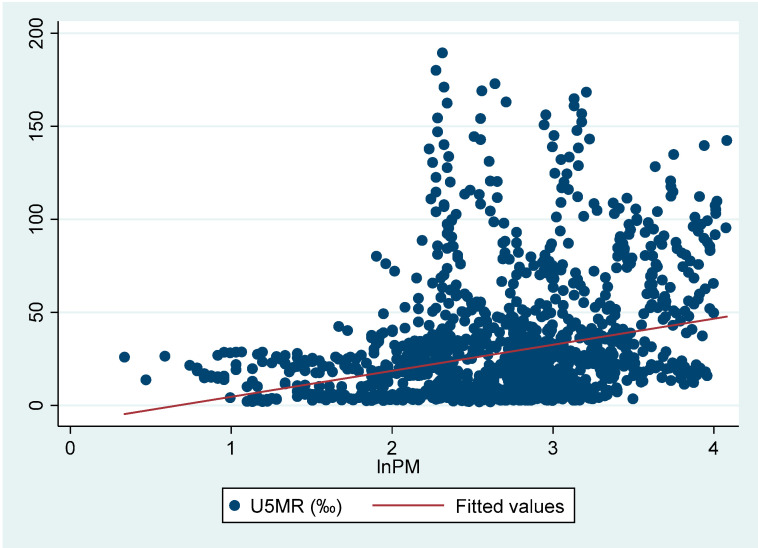
Fine particulate air pollution and U5MR.

Figure 2 depicts the relationship between fine particulate air pollution and U5MR. It shows that there exists a positive relationship between PM_2.5_ concentrations (log form) and U5MR. This indicates that PM_2.5_ pollution should have adverse influences on children’s health status. In the following sections, we used econometric techniques to investigate the children’s health effects of fine particulate air pollution in a more efficient and strict way.

### 2.4. Panel Unit Root and Cointegration Tests

In order to avoid the spurious regression problem in econometric analysis, the panel unit root test was initially adopted to test the stationarity of all dependent and independent variables. To ensure the robustness of the results, this paper utilized six types of panel tests based on common unit root hypothesis (i.e., Levin, Lin and Chu (LLC) [60], Harris and Tzavalis (HT) [61] and Breitung [62] test) and individual unit root hypothesis (i.e., Im Pesaran and Shin (IPS) [63], Fisher-Augmented Dickey Fuller (Fisher-ADF) and Fisher-Phillips Perron (Fisher-PP) [64] test). The results presented in Columns 2 to 7 of Table 2 suggested that the null hypothesis that dependent and independent variables containing a unit root could not be rejected in general, at a 10% significance level. This paper thus further implemented panel unit root tests for the first differences of each variable. As shown in Table 2 Columns 8 to 13, the hypothesis of a unit root in first differences of all the variables could be rejected in most cases. In other words, their first differences were stationary. The above tests indicated that all dependent and independent variables were integrated of order one, I(1). Therefore, the panel cointegration tests should be further implemented.

Table 3 presents the results of three types of panel cointegration tests (i.e., Kao [65], Pedroni [66,67] and Westerlu [68] panel cointegration tests). Clearly, the null hypothesis of no cointegration was robustly rejected in all cases, at a 10% significance level. Therefore, we concluded that the fine particulate air pollution and U5MR were cointegrated. In other words, there is a long-run equilibrium relationship between these variables. In the following sections, this paper further estimates the impacts of PM_2.5_ pollution on U5MR.

## 3. Results and Discussions of the Impacts of Fine Particulate Air Pollution on U5MR

### 3.1. Full Sample Results

In this subsection, we examined the impacts of fine particulate air pollution on U5MR based on the panel data of 94 countries over the period 1998–2014. Overall, there were three different econometric specifications of the panel data regression equation, i.e., ordinary least squares (OLS), fixed effects (FE), and random effects (RE) models. As shown in Table 4, the specification tests (i.e., F test and Hausman test) suggested that the FE models should be considered as the most appropriate models to investigate the relationship between PM_2.5_ pollution and U5MR. Thus, the FE estimators were employed to estimate Equation (2).

According to Table 4, the significant and positive impacts of fine particulate air pollution on U5MR can be clearly observed. As reported in Column 2 of Table 4, PM_2.5_ pollution was positively related to U5MR at the 10% significance level. In addition, the estimated coefficient of *lnPM* was 3.796. This means that if the annual average of PM_2.5_ concentrations in a country rises 1%, then the U5MR for that nation increases by 3.796/1000. These findings indicated that PM_2.5_ pollution poses a serious hazard to children’s health, which is in line with the findings of Egondi et al. [20] and Owili et al. [21]. Simultaneously, we confirmed H1.

In order to assess the robustness of our findings from the full sample, several alternative estimations were performed in this paper. First, this study re-estimates Equation (2), using the geographic-mean PM_2.5_ concentrations of each country as the alternative measure of fine particulate air pollution. According to Column 3 in Table 4, PM_2.5_ pollution still exerted positive effects on U5MR. The results turned out to be very close to those shown in the baseline (Column 2 of Table 4). 

Second, to address the potential influence of outliers in the parameter estimation, this paper re-estimated Equation (2) by winsorizing all continuous variables at 1% and 99%. The results in Column 4 of Table 4 further confirmed the positive influence of PM_2.5_ pollution on U5MR.

Finally, this paper checked whether the impacts of fine particulate air pollution on children’s health outcomes could be biased due to endogeneity, which may be caused by potential reverse causality or the fact that unobserved factors were not taken into account in the estimation framework, affecting both PM_2.5_ pollution and U5MR. Specifically, to address the potential endogeneity problem, this paper employed a fixed effects instrumental variables (FE-IV) method to re-estimate Equation (2). Following most studies [69], the one-year lagged value of the log form of population-weighted PM_2.5_ concentrations was chosen as the first instrumental variable. In addition, as developed by Lewbel [70], the third-order centered moments of the log form of population-weighted PM_2.5_ concentrations were used as the second instrumental variable in this study. The results shown in Columns 5 and 6 of Table 4 further verified the significant and adverse impacts of PM_2.5_ pollution on children’s health status, which is in line with those presented in the baseline.

With regard to the control variables of economic development level, population density, innovation capacity, and education levels all appeared to exert significant and negative impacts on U5MR. This suggested that all four factors contributed to the improvement of children’s health outcomes, which is in line with what we would expect on the basis of the economic theory. We observed that urbanization had positive effects on U5MR. Urbanization avails access to medical care and health information, which favorably influences children’s health. However, it is also generally associated with pollution and congestion, which has negative influences on children’s health outcomes [42,46]. This may be the reason why urbanization has positive effects on U5MR. Finally, we noted that the coefficient of *lnHE* was statistically positive, suggesting that health expenditure per capita has statistically negative impacts on children’s health outcomes. This is similar to the findings of Fayissa and Gutema [42], and Mohsen et al. [45]. As discussed by Fayissa and Gutema [42], the significant and negative impacts of health expenditure on children’s health status may first arise from multicollinearity (In the sample period, the correlation coefficient between the log form of GDP per capita and log form of health expenditure per capita was 0.96). In addition, the high health expenditure in one country may be generally attributed to the user fees or taxes collected from the users. In this case, the increment expenditure in health crowds out the consumption of life nurturing and sustaining goods. If the crowding-out effects exceed the positive impacts of health facility provision owing to the increased health expenditures, the health expenditure has adverse effects on health outcomes.

### 3.2. Results in Different Countries

As discussed in Section 2, the health effects of fine particulate air pollution may differ among countries at different development levels due to the great discrepancy in fine particulate air pollution levels as well as socioeconomic status. As noted already, according to the UN [16], the 94 countries selected in this paper can be classified as four types of countries. Whereas, considering the sample countries of economies in transition in this paper was relatively small (9 countries) and the socioeconomic development level of developing economies was similar to that of economies in transition, this paper combined these two types of countries. Accordingly, the sample countries were eventually classified as three types of countries, i.e., developed economies, economies in transition, and developing economies, as well as least developed countries, among which both the socioeconomic development level and PM_2.5_ concentrations notably differed. Table 5 reports the mean of U5MR and PM_2.5_ concentrations in three types of countries. On average, the highest U5MR was observed for the least developed economies (95.21‰), followed by economies in transition and developing economies (29.94‰). The U5MR of developed economies was just 6.4‰ on average, which was much lower than the other two types of countries. Meanwhile, the PM_2.5_ concentrations of the least developed countries reached 23.17 ug/m^3^ on average, followed by economies in transition and developing economies (17.98 ug/m^3^). The PM_2.5_ concentration of developed economies was 14.01 ug/m^3^, which was the lowest among the three types of countries. Hence, it was expected that the relatively poor health status of children in non-developed countries especially in the least developed countries may be attributed to the severe fine particulate air pollution to a large extent. This paper conducted empirical studies to test this in the following context.

Table 6, Table 7 and Table 8 provide the effects of fine particulate air pollution on U5MR of developed economies, economies in transition, and developing economies, and least developed countries, respectively. Clearly, the health-damaging effects of children in different types of countries notably varied. Specifically, as shown in Columns 2–5 of Table 6, every estimated coefficient of *lnPM* were not statistically significant at a 10% significance level. This suggested that the insignificant impacts of PM_2.5_ pollution on U5MR were robust to the change in the measurement of PM_2.5_ pollution and controls for potential endogeneities. However, according to Columns 2–5 of Table 7 and Table 8, we found that the influences of PM_2.5_ pollution on U5MR turned out to be statistically significant in economies in transition and developing economies, as well as least developed countries. This indicated that the adverse health effects of PM_2.5_ pollution were highly robust in both economies in transition and developing economies, as well as for the least developed countries. Additionally, the estimated coefficients of *lnPM* in the least developed countries were larger than those in economies in transition and developing economies in general (e.g., when taking into account of the baseline results, the estimated coefficients in least developed countries and economies in transition and developing economies were 12.11 and 5.767, respectively). This suggested that the adverse impacts of PM_2.5_ pollution on children’s health in least developed countries were more significant than those in economies in transition and developing economies. To sum up, the adverse health effects of fine particulate air pollution in countries with low socioeconomic development levels were larger than those with high socioeconomic development levels. Thus, H2 was confirmed.

We observed that the impacts of control variables on U5MR differed significantly in various types of countries. Specifically, both economic development and urbanization exerted significant and negative impacts on U5MR in developed countries, while the effects of population density and health expenditure per capita were significantly positive. In economies in transition and developing economies, economic development and population density had negative influences on U5MR, whereas the impacts of health expenditure per capita and education level were positive. As for least developed countries, both population density and technological innovation were important factors adversely affecting U5MR. Impacts of population density on U5MR were significantly negative in both economies in transition and developing economies, as well as for the least developed countries, while positive in developed economies. Overall, the public heath infrastructure in economies in transition and developing economies, as well as the least developed countries, was not as sound as that in developed economies. The concentration of a population can give more access to medical services under the relatively weak public heath infrastructure in economies in transition and developing economies, as well as the least developed countries, and thus has positive influences on children’s health in these countries. Additionally, the impacts of education levels on U5MR were found to be insignificant in developed economies and least developed countries, and positive in economies in transition and developing economies. Although well-educated groups pay more attention to the health status, they always experience higher levels of work stress, which contributes to the reduction of mental and physical health outcomes [71], and eventually have adverse impacts on the health outcomes of their children. This may to a certain extent explain why the effects of education levels on children’s health outcomes are not significantly positive.

## 4. Does Public Service Influence the PM_2.5_-Mortality Relationship?

### 4.1. Panel Threshold Model Setting

As noted previously, public service may be a primary factor influencing the PM_2.5_-mortality link. In order to test whether the effect of PM_2.5_ pollution on U5MR depends on public service, the panel threshold regression model developed by Hansen [72] was applied in this paper.

Clearly, if the influence of fine particulate air pollution on health outcomes is related to the performance of a country’s public education spending and sanitation service, the coefficients on PM_2.5_ pollution vary with these two factors. That is to say, there can be threshold effects (nonlinear relationship) between PM_2.5_ pollution and children’s health status. The panel threshold regression model proposed by Hansen [72], which has the advantage of exogenously checking the existence of potential threshold effects, as well as estimating the threshold values (cut-off values) based on the characteristics of the data themselves, has been widely regarded an efficient tool to capture the threshold effects in different fields [73,74,75,76]. The panel threshold model with a single threshold can be expressed as follows:

(3) $$\begin{array}{c} U5MR_{it}=c+\lambda_{1}\ln PM_{it}I(q_{it}\leq \gamma )+\lambda_{2}\ln PM_{it}I(q_{it}>\gamma )+\beta_{1}\ln Eco_{it}+\beta_{2}\ln PD_{it}+\\ \beta_{3}Urb_{it}+\beta_{4}Inno_{it}+\beta_{5}\ln Edu_{it}+\beta_{6}\ln HE_{it}+\alpha_{i}+\lambda_{t}+\varepsilon_{it} \end{array}$$

where I(⋅) stands for the indicator function. If the expression in the bracket is true, it is valued at 1. On the contrary, it is valued at 0. q and γ denote threshold variables and threshold values, respectively. In this study, threshold variables included public education spending (*PES*) and sanitation service (*SE*), which were measured by the share of public education expenditure in GDP and the percentage of population with access to improved sanitation facilities, respectively. For any given threshold value γ, the slope coefficients can be calculated and the sum of squared errors $S_{1}(\gamma )$ are obtained correspondingly. Then, the threshold value $\overset{\wedge }{\gamma }$ are obtained by minimizing $S_{1}(\gamma )$, that is: $\hat{\gamma }=\underset{\gamma }{\arg\min}S_{1}(\gamma )$.

The multiple panel threshold regression model with more than one threshold can be extended accordingly. Take double threshold as an example, if the two threshold values are $\gamma_{1}<\gamma_{2}$, the model can be described in Equation (4).

(4) $$\begin{array}{c} U5MR_{it}=c+\lambda_{1}\ln PM_{it}I(q_{it}\leq \gamma_{1})+\lambda_{2}\ln PM_{it}I(\gamma_{1}<q_{it}\leq \gamma_{2})+\lambda_{3}\ln PM_{it}I(q_{it}>\gamma_{2})+\\ \beta_{1}\ln Eco_{it}+\beta_{2}\ln PD_{it}+\beta_{3}Urb_{it}+\beta_{4}Inno_{it}+\beta_{5}\ln Edu_{it}+\beta_{6}\ln HE_{it}+\alpha_{i}+\lambda_{t}+\varepsilon_{it} \end{array}$$

Similarly, the threshold values $\gamma_{1}$ and $\gamma_{2}$ can be determined by seeking the minimum value of the sum of squared errors. For more details, please see Hansen [72].

### 4.2. Threshold Examination and Analysis

This paper first tested whether there existed a threshold effect between fine particulate air pollution and U5MR by choosing public education spending and sanitation service as threshold variables. If the null hypothesis of no threshold effect in Equation (3) was rejected, this study would then determine the number of thresholds in a multiple threshold model. As shown in Table 9, the threshold effects between PM_2.5_ pollution and U5MR were statistically significant at 10% significance level, no matter whether public education spending or sanitation service were selected as threshold variables. Specifically, the sanitation service had single threshold effects with a threshold value of 41.3%. Meanwhile, the double threshold values of public education spending were identified. These findings indicate that the influences of fine particulate air pollution on U5MR are related to a country’s public service performance.

Table 10 presents the estimated parameters for panel threshold regression. As reported in Column 2 in the table, when public education spending is chosen as the threshold variable, the size of the coefficient on fine particulate air pollution (*lnPM*) decreases with the rise of public education spending. To be more specific, when the ratio of public education expenditure in GDP was less than or equal to the first threshold (3.39%), PM_2.5_ pollution had significant and positive effects on U5MR, with a coefficient of 5.870. When public education spending lies between the first threshold and the second threshold (5.47%), the impacts of PM_2.5_ pollution on U5MR were still significantly positive. However, the estimated coefficient decreased to 3.817. Once the percentage of public education expenditure in GDP exceeded the second threshold, the estimated coefficient of *lnPM* further decreased to 2.535. The above findings reveal that increasing public expenditure on education should be an efficient way to reduce the health-damaging effects of PM_2.5_ pollution on children. According to Column 3 of Table 10, when sanitation service was selected as the threshold variable, there still existed a significant and positive relationship between PM_2.5_ pollution and U5MR in different regimes. However, the estimated coefficient of *LnPM* decreased with the improvement of sanitation services. Specifically, when the ratio of population with access to improved sanitation facilities exceeded the threshold (41.3%), the estimated coefficient of *LnPM* decreased from 8.753 to 3.402. These findings suggested that the improvement of sanitation services was conducive to reducing the adverse influences of fine particulate air pollution on the health outcomes of children. To summarize, public service plays significant roles in moderating the PM_2.5_-mortality link. Thus, H3 was confirmed.

Table 11 further reports the ratio of countries that fall into a particular regime of the two threshold variables. With regard to public education spending, it is observed that about 30% of the observations during 1998–2014 were below the first threshold value, the overwhelming majority of which were economies in transition and developing economies, as well as the least developed countries. Nearly half of the observations were between the first threshold value and the second threshold value, and about 20% of the observations were above the second threshold value. In terms of sanitation service, it was apparent that only about 10% of the observations were below the threshold value, most of which were the least developed countries. In particular, there were eight countries where the percentage of population with access to improved sanitation facilities were still less than 41% in 2014. Except for India and Kenya, the other six countries (i.e., Cambodia, Congo, Ethiopia, Mozambique, Sudan, and Tanzania) were least developed countries.

As stated above, developed countries offer a higher quality of public service in general, which can explain why the children’s health-damaging effects of fine particulate air pollution in developed economics are less significant than those in the other two types of countries, particularly in the least developed countries.

## 5. Conclusions

Although a great number of studies have investigated the health effects of PM_2.5_ pollution at the individual level in a given developed country or non-developed large country, the research in non-developed countries, especially the least developed countries, is still rare. Meanwhile, very few studies have paid attention to exploring the factors in alleviating the adverse health impacts of PM_2.5_ pollution. This paper took into account the large discrepancy in socioeconomic development levels among different countries and proposed the research hypotheses that PM_2.5_ pollution had positive effects on U5MR globally but the effects in the countries with low socioeconomic development levels were larger than those with high socioeconomic development levels. In addition, considering children’s health effects of PM_2.5_ pollution should be related to the condition of a country’s public service, this study further put forwards the hypothesis that the effects of PM_2.5_ pollution on U5MR decreased with the rise of a country’s public education spending and sanitation service improvement. Using panel data of 94 countries from 1998 to 2014, this paper verified these three hypotheses and provided a deeper understanding regarding the health effects of PM_2.5_ pollution. More specifically, the main findings can be summarized as follows.

Firstly, fine particulate air pollution had significant and positive influences on U5MR globally. Meanwhile, the impacts were robust to the change in the measurement of fine particulate air pollution, as well as controls for possible outliers and potential endogeneities.

Secondly, the statistical significance and magnitude of the effects showed heterogeneity among various types of countries under different development levels. Specifically, the impacts of PM_2.5_ pollution on U5MR were significantly positive in both economies in transition and developing economies, as well as the least developed countries, while insignificant in developed economies. In addition, the adverse impact of PM_2.5_ pollution on children’s health outcomes in the least developed countries was more significant than that in economies in transition and developing economies. 

Thirdly, public services reflected by public education spending and sanitation service played a positive moderating role in the PM_2.5_-mortality link. The effects of fine particulate air pollution on U5MR decreased with the rise of a country’s public education spending and sanitation service improvement. To be more specific, when the ratio of public education expenditure in GDP of a country exceeded the first threshold value 3.39% and the second threshold value 5.47%, the magnitude of the impacts of PM_2.5_ pollution on U5MR significantly decreased accordingly. When the percentage of population with access to improved sanitation facilities in a country is over 41.3%, the health damaging effects were reduced by more than half.

Several important policy implications can be drawn from this study. First, this study provided clear evidence for the adverse effects of fine particulate air pollution on children’s health globally and the impacts in economies in transition and developing economies, as well as the least developed countries were statistically significant. Policymakers, especially those for non-developed countries with higher PM_2.5_ concentrations, should strive to decrease the PM_2.5_ pollution to reduce the U5MR accordingly. Meanwhile, we noted that severe PM_2.5_ pollution in these countries can be attributed to the extensive economic growth mode [12,44]. Therefore, in addition to strict environmental protection policy, different policies and measures such as optimizing the industrial structure, accelerating technological innovation, and promoting renewable energy development should be jointly implemented for these countries to decrease PM_2.5_ concentrations [24,75,77,78,79,80]. Additionally, this paper demonstrated the positive role of public service in alleviating the adverse impacts of fine particulate air pollution on children’s health outcomes. Accordingly, in order to reduce the children’s health-damaging effects of PM_2.5_ pollution, the authorities, especially those of least developed countries with poor public service performance, should make efforts to increase investment in public education to widen people’s environmental and health knowledge, and perfect public sanitation service systems for prevention and treatment of the diseases caused by PM_2.5_ pollution.

There are still some limitations in this study. For example, although PM_2.5_ concentrations estimated by satellite observations fit well for a dataset collected from monitoring stations, there still exists large uncertainty in parts of Asia, Africa, and Latin America due to sparse ground-based monitoring and challenging conditions for retrieval and simulation [3,57]. The uncertainty in the PM_2.5_ estimation may affect the empirical results to some extent. As part of future research, considering there may exist large differences in PM_2.5_ pollution among different regions of some big countries such as China, we plan to investigate the health effects of PM_2.5_ pollution in a specific country at the province or city scale.

## Figures and Tables

**Table 1:** Descriptive statistics.

Symbol	Definition	Unit	Mean	Std	Min	Max
*U5MR*	Under-five mortality rate	‰	27.87	32.32	2.0	189.5
*lnPM*	Log form of annual PM_2.5_ concentrations	ug/m^3^	2.66	0.62	0.34	4.08
*lnEco*	Log form of GDP per capita	dollar	8.84	1.49	5.23	11.61
*lnPD*	Log form of population density	kilometer	4.25	1.25	0.89	8.95
*Urb*	Urbanization level	%	61.20	20.91	8.55	100
*Inno*	Ratio of RD expenditure to GDP	%	0.86	0.89	0.00	4.29
*lnEdu*	Log form of average schooling years	year	2.07	0.44	0.34	2.57
*lnHE*	Log form of health expenditure per capita	dollar	6.07	1.68	2.25	9.18

**Table 2:** Results of panel unit root test.

	Levels						Diff					
	LLC	HT	Breitung	IPS	ADF	PP	LLC	HT	Breitung	IPS	ADF	PP
*U5MR*	−2.94 (0.02)	0.72 (1.00)	3.29 (1.00)	9.32 (1.00)	306.3 (<0.01)	403.0 (<0.01)	−11.81 (<0.01)	−0.02 (<0.01)	2.54 (0.99)	−3.66 (<0.01)	183.3 (0.58)	449.5 (<0.01)
*lnPM*	2.49 (0.99)	0.03 (<0.01)	0.66 (0.75)	−3.57 (<0.01)	353.4 (<0.01)	928.9 (<0.01)	−7.24 (<0.01)	−0.37 (<0.01)	−4.13 (<0.01)	−17.1 (<0.01)	742.2 (<0.01)	2995.6 (<0.01)
*lnEco*	0.51 (0.70)	0.80 (1.00)	1.46 (0.93)	3.20 (1.00)	197.4 (0.30)	275.9 (<0.01)	−6.81 (<0.01)	0.33 (<0.01)	−3.86 (<0.01)	−7.40 (<0.01)	273.5 (<0.01)	844.1 (<0.01)
*lnPD*	1.83 (0.97)	0.84 (1.00)	−1.31 (0.09)	0.45 (0.67)	158.3 (0.94)	417.8 (<0.01)	−4.14 (<0.01)	0.41 (<0.01)	0.65 (0.74)	−3.92 (<0.01)	286.8 (<0.01)	455.6 (<0.01)
*Urb*	17.8 (1.00)	0.76 (1.00)	4.91 (1.00)	4.63 (1.00)	593.9 (<0.01)	1301.2 (<0.01)	4.03 (1.00)	0.67 (<0.01)	6.35 (1.00)	−1.74 (0.04)	1019.9 (<0.01)	700.2 (<0.01)
*Inno*	−5.09 (<0.01)	0.59 (0.29)	−0.88 (0.19)	−1.93 (0.03)	171.3 (0.80)	259.9 (<0.01)	−10.92 (<0.01)	−0.10 (<0.01)	−4.07 (<0.01)	−11.2 (<0.01)	318.2 (<0.01)	1714.8 (<0.01)
*lnEdu*	−1.87 (0.03)	0.87 (1.00)	−2.51 (0.01)	−0.85 (0.20)	209.2 (0.14)	164.6 (0.89)	−10.64 (<0.01)	0.61 (<0.01)	−0.78 (0.22)	−5.79 (0.05)	176.5 (0.72)	329.9 (<0.01)
*lnHE*	−2.65 (<0.01)	0.67 (0.99)	0.64 (0.74)	−1.76 (0.04)	363.4 (<0.01)	259.9 (<0.01)	−8.92 (<0.01)	0.29 (<0.01)	−3.04 (<0.01)	−15.68 (<0.01)	359.5 (<0.01)	946.9 (<0.01)

Note: “Levels” and “Diff” denote the panel unit root tests for a unit root in levels and first differences, respectively. The *p*-values are shown in parentheses. *U5MR*, *PM*, *Eco*, *PD*, *Urb*, *Inno*, *Edu,* and *HE* stand for under-five mortality rate, PM_2.5_ pollution, economic development level, population density, urbanization level, innovation capacity, educational levels and health expenditure, respectively.

**Table 3:** Results of panel cointegration test.

Types of Tests for Cointegration	Types of Test Statistics	Statistic	*p*-Value
Kao test	Modified DF t	6.155	<0.01
	DF t	1.952	0.026
	ADF t	4.556	<0.01
	Unadjusted DF t	3.609	<0.01
	Unadjusted DF t	−1.395	0.082
Pedroni test	Modified PP t	16.020	<0.01
	PP t	−16.049	<0.01
	ADF t	−14.319	<0.01
Westerlund test	Variance ratio	3.830	<0.01

Note: DF, ADF, and PP stand for Dickey Fuller, Augmented Dickey Fuller, and Phillips Perron, respectively.

**Table 4:** Estimation result in the full sample.

Variables	Dependent Variable: Under-Five Mortality Rate (U5MR, ‰)				
	FE (Baseline)	FE (Alterative Measurement)	FE (Winsorization)	FE-IV1	FE-IV2
*lnPM*	3.796 ***	3.984 ***	2.308 **	22.37 ***	10.97 ***
	(3.38)	(4.02)	(2.05)	(4.39)	(5.44)
*lnEco*	−30.99 ***	−30.84 ***	−27.95 ***	−35.13 ***	−33.00 ***
	(−21.50)	(−21.68)	(−15.61)	(−17.11)	(−21.52)
*InPD*	−101.7 ***	−102.0 ***	−108.1 ***	−108.2 ***	−106.2 ***
	(−29.62)	(−29.82)	(−32.81)	(−23.11)	(−29.24)
*Urb*	0.174 *	0.168 *	0.197 **	−0.068	0.092
	(1.76)	(1.71)	(2.03)	(−0.56)	(0.91)
*Inno*	−4.376 ***	−4.288 ***	−4.634 ***	−5.162 ***	−4.603 ***
	(−5.05)	(−4.96)	(−5.25)	(−5.44)	(−5.23)
*lnEdu*	−15.28 ***	−14.63 ***	−7.115 **	−7.212 *	−12.90 ***
	(−4.26)	(−4.07)	(−2.08)	(−1.66)	(−3.51)
*lnHE*	4.880 ***	4.895 ***	1.579 *	6.293 ***	5.112 ***
	(9.48)	(9.53)	(1.91)	(9.29)	(9.75)
Constant	713.3 ***	712.3 ***	720.8 ***	729.4 ***	729.0 ***
	(38.18)	(38.34)	(37.47)	(31.49)	(37.81)
Year dummy	Yes	Yes	Yes	Yes	Yes
F test	126.16 ***	127.27 ***	126.73 ***		
Hausman test	688.68 ***	727.50 ***	1009.07 ***		
N	1598	1598	1598	1504	1598
R^2^	0.708	0.709	0.697	0.639	0.700

Note: ***, **, and * represent significance levels of 1%, 5%, and 10%, respectively. t statistic values are shown in parentheses. Column 3 presents the estimation results of fixed effects model where geographic-mean PM_2.5_ concentrations are used to measure fine particulate air pollution. Column 4 presents the estimation results of fixed effects model where all the continuous variables are winsorized at 1% and 99%. Column 5 and Column 6 present the estimation results of fixed effect instrumental variables model, where one-year lagged value of the log form of PM_2.5_ concentrations and the third-order centered moments of the log form of PM_2.5_ concentrations are chosen as the instrumental variables, respectively. *PM*, *Eco*, *PD*, *Urb*, *Inno*, *Ed,u* and *HE* stand for PM_2.5_ pollution, economic development level, population density, urbanization level, innovation capacity, educational levels, and health expenditure, respectively.

**Table 5:** Mean of under-five mortality and PM_2.5_ concentrations in different types of countries.

	Under-Five Mortality Rate (‰)	PM_2.5_ Concentrations (ug/m^3^)
Developed economies	6.40	14.01
Economies in transition and developing economies	29.94	17.98
Least developed countries	95.21	23.17

**Table 6:** Estimation result of developed economies.

Variables	Dependent Variable: Under-Five Mortality Rate (U5MR, ‰)			
	FE (Baseline)	FE (Alterative Measurement)	FE-IV1	FE-IV2
*lnPM*	−0.131	0.184	0.606	−0.063
	(−0.40)	(0.64)	(0.31)	(−0.10)
*lnEco*	−9.081 ***	−9.035 ***	−8.118 ***	−9.069 ***
	(−13.69)	(−13.65)	(−10.96)	(−13.52)
*InPD*	9.220 ***	9.214 ***	9.282 ***	9.228 ***
	(7.85)	(7.85)	(7.25)	(7.84)
*Urb*	−0.0515 *	−0.0496 *	−0.0397	−0.0509 *
	(−1.85)	(−1.79)	(−1.15)	(−1.79)
*Inno*	0.0726	0.0703	0.0301	0.0715
	(0.41)	(0.39)	(0.17)	(0.40)
*lnEdu*	0.0978	0.150	0.510	0.104
	(0.08)	(0.13)	(0.41)	(0.09)
*lnHE*	1.010 ***	1.018 ***	0.711 ***	1.011 ***
	(3.97)	(4.00)	(2.70)	(3.97)
Constant	57.30 ***	55.76 ***	43.03 **	56.90 ***
	(4.88)	(4.79)	(2.52)	(4.65)
Year dummy	Yes	Yes	Yes	Yes
F test	132.79 ***	131.80 ***		
Hausman test	108.07 ***	113.36 ***		
N	612	612	576	612
R^2^	0.816	0.816	0.804	0.816

**Table 7:** Estimation result of economies in transition and developing economies.

Variables	Dependent Variable: Under-Five Mortality Rate (U5MR, ‰)			
	FE (Baseline)	FE (Alterative Measurement)	FE-IV1	FE-IV2
*lnPM*	5.767 ***	5.410 ***	28.27 ***	9.643 ***
	(5.40)	(5.97)	(4.02)	(5.32)
*lnEco*	−20.41 ***	−20.24 ***	−20.50 ***	−20.81 ***
	(−12.03)	(−11.99)	(−8.95)	(−12.11)
*InPD*	−70.81 ***	−70.93 ***	−78.67 ***	−73.26 ***
	(−16.06)	(−16.18)	(−11.82)	(−16.13)
*Urb*	−0.0159	−0.0122	−0.309 **	−0.063
	(−0.18)	(−0.14)	(−2.17)	(−0.68)
*Inno*	−0.756	−0.599	−2.062 *	−0.962
	(−0.78)	(−0.62)	(−1.65)	(−0.98)
*lnEdu*	12.54 ***	13.10 ***	31.09 ***	15.33 ***
	(3.52)	(3.68)	(4.56)	(4.09)
*lnHE*	3.061 ***	3.080 ***	4.138 ***	3.095 ***
	(5.44)	(5.50)	(4.98)	(5.45)
Constant	449.6 ***	449.1 ***	395.9 ***	450.3 ***
	(17.33)	(17.38)	(11.07)	(17.20)
Year dummy	Yes	Yes	Yes	Yes
F test	186.19 ***	190.78 ***		
Hausman test	198.37 ***	210.89 ***		
N	816	816	768	816
R^2^	0.741	0.743	0.557	0.737

**Table 8:** Estimation result of least developed countries.

Variables	Dependent Variable: Under-Five Mortality Rate (U5MR, ‰)			
	RE (Baseline)	RE (Alterative Measurement)	RE-IV1	RE-IV2
*lnPM*	12.11 *	12.97 *	21.56 **	28.56 ***
	(1.71)	(1.70)	(2.02)	(2.80)
*lnEco*	−3.258	−3.224	−10.59 *	−9.468
	(−0.56)	(−0.56)	(−1.70)	(−1.47)
*InPD*	−42.85 ***	−41.85 ***	−43.01 ***	−47.69 ***
	(−5.49)	(−5.47)	(−5.64)	(−5.82)
*Urb*	−0.422	−0.472	−0.555	−0.727
	(−0.75)	(−0.82)	(−1.01)	(−1.24)
*Inno*	−18.18 ***	−17.68 ***	−17.42 ***	−18.18 ***
	(−3.82)	(−3.71)	(−3.64)	(−3.75)
*lnEdu*	−6.103	−5.700	−0.829	−0.460
	(−0.59)	(−0.55)	(−0.08)	(−0.04)
*lnHE*	−1.242	−1.198	−0.963	−1.041
	(−0.88)	(−0.84)	(−0.57)	(−0.72)
Constant	302.8 ***	298.1 ***	267.3 ***	310.5 ***
	(5.68)	(5.62)	(4.78)	(5.75)
Year dummy	YES	YES	YES	YES
F test	38.01 ***	37.36 ***		
Hausman test	31.23	21.82		
N	170	170	160	170

Note: ***, **, and * represent significance levels of 1%, 5%, and 10%, respectively. t statistic values are shown in parentheses. Column 3 presents the estimation results of random effects model where geographic-mean PM_2.5_ concentrations is used to measure fine particulate air pollution. Column 4 and Column 5 present the estimation results of random effect instrumental variables model, where one-year lagged value of the log form of PM_2.5_ concentrations and the third-order centered moments of the log form of PM_2.5_ concentrations are chosen as the instrumental variables, respectively.*PM*, *Eco*, *PD*, *Urb*, *Inno*, *Edu,* and *HE* stand for PM_2.5_ pollution, economic development level, population density, urbanization level, innovation capacity, educational levels and health expenditure, respectively.

**Table 9:** Tests for threshold effects between fine particulate air pollution and U5MR.

Threshold Variable	No. of Thresholds	F-Value	*p*-Value	Threshold Estimates	95% Confidence Interval
Public education spending	Single	95.39 ***	0.002	3.39%	[3.37%, 3.41%]
	Double	37.18 *	0.066	5.47%	[5.40%, 5.49%]
	Triple	29.12	0.332	4.24%	[4.20%, 4.25%]
Sanitation service	Single	141.06 **	0.010	41.3%	[41.1%, 41.9%]
	Double	40.34	0.456	70.5%	[69.3%, 70.8%]

Note: ***, **, and * represent significance levels of 1%, 5%, and 10%, respectively; F-value, *p*-value and 95% confidence interval are the results of the bootstrap simulation for 500 times.

**Table 10:** Threshold regression estimation results.

Coefficients	Public Education Spending as Threshold Variable	Sanitation Service as Threshold Variable
*lnPM I*(*PES*_it_ ≤ 3.39%)	5.870 *** (5.34)	
*lnPM I*(3.39% < *PES*_it_ ≤ 5.47%)	3.817 *** (3.53)	
*lnPM I*(5.47% < *PES*_it_)	2.535 ** (2.32)	
*lnPM I*(*SE*_it_ ≤ 41.3%)		8.581 *** (7.45)
*lnPM I*(41.3% < *SE*_it_)		3.432 *** (3.19)
*lnEco*	−32.52 *** (−23.25)	−31.27 *** (−22.64)
*lnPO*	−97.78 *** (−29.30)	−94.39 *** (−28.16)
*Urb*	0.207 ** (2.18)	0.152 (1.61)
*lnno*	−4.050 *** (−4.85)	−3.346 *** (−4.01)
*lnEdu*	−15.47 *** (−4.47)	−14.83 *** (−4.31)
*lnHE*	5.171 *** (10.42)	4.606 *** (9.33)
Constant	705.3 *** (39.18)	685.2 *** (37.93)
Year dummy	Yes	Yes
N	1598	1598
R^2^	0.730	0.732

Note: ***, ** represent significance levels of 1% and 5%, respectively. t statistic values are shown in parentheses. *PES*, *SE*, *PM*, *Eco*, *PD*, *Urb*, *Inno*, *Edu,* and *HE* stand for public education spending, sanitation service, PM_2.5_ pollution, economic development level, population density, urbanization level, innovation capacity, educational levels, and health expenditure, respectively.

**Table 11:** Proportion of countries in each threshold variable regime.

Threshold Variables	Regime	Ratio of Countries in Each Regime
Public education spending	*PES* ≤ 3.39%	26.35%
	3.39% < *PES* ≤ 5.47%	50.25%
	5.47% < *PES*	23.40%
Sanitation service	*SE* ≤ 41.3%	10.89%
	41.3% < *SE*	89.11%
